# A scoping review of mental health literacy in performing and creative artists: identifying current gaps and future directions

**DOI:** 10.3389/fpsyg.2025.1329029

**Published:** 2025-08-22

**Authors:** Shelly-Anne Li, Naoko Sakata, Gemma Donn

**Affiliations:** ^1^Department of Family & Community Medicine, Temerty Faculty of Medicine, University of Toronto, Toronto, ON, Canada; ^2^The Al & Malka Green Artists' Health Centre, University Health Network, Toronto, ON, Canada; ^3^Faculty of Health Sciences, School of Rehabilitation Sciences, University of Ottawa, Ottawa, ON, Canada

**Keywords:** mental health literacy, scoping review, artists, mental health, performing arts

## Abstract

**Introduction:**

Mental health literacy is a multifaceted construct that consists of helping individuals recognize the early warning signs of mental health conditions, understanding the concept of stigma and misconceptions associated with mental illness, encouraging appropriate help-seeking behaviors, and facilitating access to mental health services. However, mental health literacy remains a largely unexplored topic in artists’ health literature. This scoping review examines the conceptualization, operationalization, and measurement of mental health literacy in performing and creative artists.

**Methods:**

We conducted a comprehensive search across multiple databases, including MEDLINE, CINAHL, PubMed, EMBASE, PsycINFO, Web of Science, and Cochrane. Our search was designed to identify articles relevant to mental health literacy among artists, encompassing aspects related to the understanding, identification, and education of mental health conditions. Two independent reviewers conducted both abstract and full-text screenings. Our findings are synthesized using the four components of mental health literacy as a framework for organization.

**Results:**

Of the 669 unique citations, 26 articles met the inclusion criteria; of these, 23 focused on performing artists. The articles were published between 1997 and 2024, with at least 4,710 participants from nine countries. Only one study included a definition of mental health literacy. Sixteen articles included one of the four components of mental health literacy, nine included two, four included three, and one had all four components.

**Discussion:**

Despite the high prevalence of mental health challenges among performing and creative artists, there is a disproportionately low number of interventions aimed at increasing mental health literacy compared to other fields, such as sports medicine and education. This highlights the need for more comprehensive efforts to increase awareness and understanding of mental health issues among artists. Furthermore, the lack of consensus on the conceptualization, operationalization, and measurement of mental health literacy in this field prompts further research. A standardized definition and validated instrument could facilitate more robust research on mental health literacy in the artists’ health literature and help identify effective interventions. Future research can build on this review to develop and evaluate interventions aiming to improve mental health literacy in artists.

## Introduction

Performing and creative artists, such as actors, sculptors, painters, musicians, acrobats, and dancers; are at an increased risk of mental health challenges, including depression, performance anxiety, burnout, and substance abuse, compared to the general population (van den Eynde et al., 2016; Power et al., 2015). For instance, individuals in creative industries have experienced rates of depression five times greater (van den Eynde et al., 2016) and were treated for mental health problems more frequently than the general population (Kyaga et al., 2011, 2013). Among musicians, higher symptoms of both anxiety and depression were reported when compared to the general population, as demonstrated in Norwegian (Détári et al., 2020; Vaag et al., 2016) and United Kingdom (UK; Gross and Musgrave, 2016) samples. Specifically, musicians’ tendency to have maladaptive perfectionism (excessive concerns about being evaluated) is significantly correlated with anxiety, which leads to heightened risks of long-term mental health challenges (Alpheis and Altenmüller, 2024). Heightened risks of mental health challenges in the artistic population have been associated with a range of contextual factors unique to the arts industry, including the intense pressure to perfect their artistic practice (Dwarika and Haraldsen, 2023; Patston and Osborne, 2016; Pecen et al., 2018), the social isolation and rejection that can come with the industry (Duarte, 2020), financial precarity (King et al., 2019; Loveday et al., 2023), and the inevitable scrutiny and negative evaluation of their practice (Osborne et al., 2005). In addition, a recent study using a social-ecological model to map the health determinants of artists found that artists’ identities are deeply intertwined with their creative work. Their sense of self-worth is often closely linked to their artistic productivity, making them particularly vulnerable to mental health challenges during periods when they are unable to create or encounter setbacks in their artistic careers (Li et al., 2024). Perhaps more concerning is the additional evidence suggesting that mental health problems are already prominently reported among college and university arts students (Dempsey and Comeau, 2019; Kenny et al., 2014) or even earlier, particularly for performing artists, during training in adolescence and childhood (Anderson, 2011; Kaleńska-Rodzaj, 2020).

Despite the prevalence of mental health challenges in performing and creative artists, help-seeking behaviors in this subpopulation have been reportedly low. A recent study found that help-seeking behaviors are even lower among musicians who have greater depressive symptoms (Rosenbaum and Weibelzahl, 2023). Researchers in sports medicine have long recognized that (i) one of the main barriers to seeking mental health treatment is low mental health literacy (Castaldelli-Maia et al., 2019), and (ii) mental health literacy interventions are effective in increasing help-seeking behaviors and reducing self-stigma among athletes (Breslin et al., 2018; Bu et al., 2020; Gorczynski et al., 2021; Kern et al., 2017). In school settings, mental health literacy interventions are frequently embedded in educational curricula to decrease stigmatizing attitudes and support early detection of mental health disorders (Kutcher et al., 2013; Mcluckie et al., 2014; Merritt et al., 2007). In one of the few studies that examined mental health literacy in performing artists, investigators found that musicians with lower mental health literacy experienced higher levels of performance-related health issues (Guptill et al., 2022). Researchers recognize the importance of educating performing and creative artists about mental health and enhancing help-seeking attitudes to prevent or manage mental health conditions; however, the topic of mental health literacy is rarely addressed in the artists’ health literature.

Mental health literacy is an evolving concept. Since 2000, it is considered a multifaceted construct that comprises of four major components: (i) recognition of mental health disorders, (ii) knowledge of their risk factors and of seeking mental health information and support services, (iii) attitudes that promote recognition and appropriate help-seeking; and (iv) understanding the concept of stigma, which promotes self-help strategies and help-seeking efficacy (Jorm, 2000; Sampaio et al., 2022). The recognition of mental health risks and their symptoms in artists are critical to their well-being, since awareness and appraisal of the problem allow for healthy help-seeking behaviors (Rickwood et al., 2005). It is particularly important for artists to understand the symptoms of mental health problems that are common in their discipline (Cardinal and Hilsendager, 1997). Knowledge of risk factors can be used to help prevent mental health problems, and it may also influence decisions related to seeking help and uptake of treatment (Jorm, 2012). Similarly, knowledge related to information and support services may also impact the individual’s ability to receive treatment and respond to treatment instructions (Jorm, 2000). An artist with adequate mental health literacy may be more likely to recognize the early warning signs of a mental health condition, such as depression or anxiety, and seek help and support before the condition becomes more severe. They may also be more likely to understand how to manage and cope with the stress and pressure that comes with the industry, which can help prevent the onset of mental health challenges and reduce the stigma surrounding mental health conditions (Bennett et al., 2023).

While the existing literature in other fields, such as sports medicine and education, demonstrates the importance of mental health literacy and has operationalized this concept, its status in artists’ health literature remains unclear. The objectives of this scoping review are to identify the literature on mental health literacy in the artists’ health discipline, clarify how mental health literacy is conceptualized, operationalized, and measured in artists’ health literature, and summarize our main findings of these publications. Conceptualization involves defining or specifying the meaning of mental health literacy. Different researchers may conceptualize a concept slightly differently. Operationalization is the process by which a researcher precisely specifies how a construct will be empirically investigated. Operationalization works by recognizing specific indicators that are measurable and/or observable in research. Measurement represents the process of observing and recording the observations that are collected as part of the inquiry (Jiao et al., 2022).

### Research questions

The following research questions (RQ) are used to guide our scoping review:

*RQ1:* What types of research designs, methods, publication sources, and populations are conducted in the research on mental health literacy in the artists’ health literature?

*RQ2:* How has mental health literacy been conceptualized, operationalized and measured within the artists’ health literature?

To address the research questions, we developed a protocol (Moher et al., 2015) in alignment with the objectives of a scoping review, which aims to delineate the extent or range of a body of literature on a specific topic, elucidate research methodologies employed, and provide an overview of existing literature (Munn et al., 2018). Table 1 presents the definitions and parameters that guided our approach to conceptualize, operationalize, and identify measures of mental health literacy within the context of our scoping review. We also used the Preferred Reporting Items for Systematic reviews and Meta-Analyses extension for Scoping Reviews (PRISMA-ScR) Checklist (Tricco et al., 2018) to guide our scoping review protocol development and reporting (Appendix 1).

**Table 1:** Description of key terms and parameters for data extraction.

Term	Operational definition	Parameters for data extraction to inform understanding of mental health literacy
Conceptualization	Assigning meaning to something	Definition of mental health literacy, and main components of mental health literacy
Operationalization	Selecting observable phenomena to represent abstract concepts	Dimensions and indicators of mental health literacy Based on our indicators, what questions were asked to represent mental health literacy, what observations were made, what specific attributes are measured?
Measurement	Process of observing and recording observations, or assigning numbers to a phenomenon	Level of measurement such as nominal, ordinal, interval or ratio and type of measures such as survey, scaling, qualitative, used for mental health literacy

## Methods

### Databases searched

We sought relevant articles pertaining to mental health literacy in artists, including the understanding, identification, and treatment of mental health conditions. MEDLINE, EMBASE, PubMed, CINAHL, Web of Science and PsycINFO databases were searched from database inception to week 1 of June 2025.

### Search strategy

A search strategy was created in a consultation meeting with a University of Toronto librarian. We followed the Peer Review of Electronic Search Strategies (PRESS; McGowan et al., 2016) guidelines to optimize sensitivity and specificity of our search results. The search strategy for MEDLINE (see Appendix 2) was adapted to PubMed, PsycINFO, and EMBASE databases. The strategy included separate terms related to artistic professions, as well as health literacy, mental wellness, and a variety of psychiatric illnesses.

### Eligibility criteria of included studies

We included publications that (i) investigated artists, art workers, art students, or art educators; (ii) assessed mental health literacy, mental health education, and/or mental health promotion; and in English language. Articles could be of any study design or publication (peer-reviewed articles, conference abstracts, protocols, dissertations and theses, and book chapters). We excluded publications that: (i) reported only on the prevalence, etiology, treatment or assessment of any mental disorder classified in the Diagnostic and Statistical Manual of Mental Disorders Fifth Edition Text Revised (DSM-5-TR), (ii) investigated or described the use of art to improve health and/or to promote health education of individuals who are not creative or performing artists, (iii) reported only on the physical health of creative or performing artists, (iv) are not written in English language, and (v) are not peer-reviewed.

### Study selection

The database search produced 625 unique citations (Appendix 3: PRISMA 2020 flow diagram). Two investigators (SAL, NS) independently screened the title and abstracts for inclusion. Discrepancies were resolved in two consensus meetings. An additional 44 papers with relevant titles and abstracts were found through handsearching of the reference list of included articles eligible for full-text screening (*n* = 53). Three papers could not be retrieved. Of the 50 articles available, 26 remained upon completion of the review process (see Appendix 3 for reasons for exclusion).

### Data abstraction

To extract data from the included studies, we used the following categories: first author and setting, study design, sample, data collection methods, main findings, and specifically noted whether the included studies had a definition of mental health literacy.

### Analysis and presentation of results

Narrative summaries were included to complement the tabular results, and we discussed how the findings relate to the research question and objectives (Khalil et al., 2021). For all parameters of investigation (conceptualization, operationalization and measurement) in this review, we used the existing components of mental health literacy (Jorm, 2000) as a guiding framework of our analysis. In addition to this descriptive narrative summary, we conducted a thematic analysis of the literature using qualitative description (Sandelowski et al., 2009) for the operationalization parameter of mental health literacy. We used thematic analysis specifically for this parameter because it involves identifying concrete indicators aligned with the four established components of mental health literacy. This level of detail benefits from thematic analysis to systematically categorize how components were applied across studies. In contrast, data within the conceptualization measurement parameters of the included studies were more varied and straightforward (e.g., whether they used an individual interview or questionnaire to assess mental health literacy constructs), respectively, making them better suited to remain as descriptive narrative syntheses rather than thematic categorization. In the thematic analysis, we (i) applied a within-case approach, which included a synthesis of the main findings of all studies identified, (ii) identified and developed descriptive themes and categories across the studies, (iii) summarized and developed overarching main findings within topics from a between-case approach (i.e., categories), and (iv) meta-analyzed the findings to answer the research questions (Dwarika and Haraldsen, 2023). The findings were categorized in accordance with an understanding of mental health as a complete and dynamic state (Keyes, 2002; Lazarus and Folkman, 1984). The thematic analysis followed the guidance of Clarke and Braun (2013).

Specifically, two coders independently read the included studies twice to familiarize themselves with the articles, and made written notes about the main points of each article. Next, an initial codebook was developed based on the four pillars of mental health literacy as a guiding framework, which contained each code and its definition, with at least one illustrative quote that represented the concept of the code. Coding and re-recoding was an iterative process that involved coders moving back and forth throughout the dataset to explore the breadth of mental health literacy and depth (how it was being operationalized). As coding progressed, two consensus meetings were held between the two coders to refine the codes across the included studies. In alignment with the qualitative description, coders stayed close to the report of each included study to ensure that codes were descriptive (semantic), capturing obvious surface meanings in the data rather than interpretative (latent) that captures implicit meaning.

We used the 15-point checklist of criteria for thematic analysis (Clarke and Braun, 2013) to ensure rigour in collating and summarizing the results. NVivo 12 Pro was used to organize the abstracted data into themes and codes, which were further explored in the results section of this review. Findings were organized into thematic categories including methodological design and key findings, but also by categories that specifically highlighted the theoretical and operational linkages such as context, conceptual and operational features, and measurements used.

### Consultation with stakeholders

We consulted with 8 knowledge stakeholders, who were either leaders in the research and practice of performing arts medicine, performing and creative artists in Canada and the United States; or practitioners who specialize in providing care to artists of all disciplines. We asked whether they knew of mental health literacy documents, literature, or peer-reviewed studies that pertain to artists. None of the stakeholders have identified any information regarding this topic. Findings from this scoping review will be returned to these stakeholders.

## Results

### Characteristics of included studies

Supplementary Table 1 presents the characteristics of the 26 included studies. The studies were published between 1997 and 2024 and were based in nine countries. At least 4,710 participants were included in the review. At least 32% (*n* = 1,502) of the participants included in this review were musicians, followed by participants in artistic disciplines such as dance (*n* = 608; 13%), circus art (*n* = 87; 1.8%), writing (*n* = 87; 1.8%), and visual art (*n* = 276; 6%). Most studies included students as the participants (*n* = 3,848; 82%), as the purpose of these studies was to evaluate strategies of health promotion and injury prevention in secondary and post-secondary education programs. Two of the studies included participants who were in administrative and leadership roles at these educational institutions, while 6 (23%) studies included participants who identified as professional working artists. Out of the 26 articles, eight referenced interventions for Music Performance Anxiety (MPA). These interventions included physical techniques such as deep breathing exercises, workshops during which students would perform for one another, instruction on coping mechanisms such as time management skills and self-care techniques, and group education programs.

### Conceptualizing mental health literacy

Only one study included a definition of mental health literacy (Barnett et al., 2019), and it conceptualized mental health literacy as knowledge of mental health issues and services. The included studies that conceptualized at least one component of mental health literacy sampled dancers, musicians, visual artists, circus artists, and novelists. Thirteen articles (46.2.%) included one of the four components (Atkins, 2009; Barnett et al., 2019; Bartos et al., 2022a, 2022b; Cardinal and Hilsendager, 1997; Griffith, 2023; Guptill et al., 2022; Harrison et al., 2019; Kaufman et al., 1996; Qu, 2022; Smith, 2021; Stuckey et al., 2021; Torres-McGehee et al., 2011; Walker and Nordin-Bates, 2010), eight (30.7%) included two components (Barton and Feinberg, 2008; Cardinal et al., 2020; Clements-Cortés et al., 2024; Ginsborg et al., 2012; Moore et al., 2024; Shaw et al., 2020; Uriegas et al., 2024; Žilinskas and Lesinskienė, 2023), four (15.4%) had three components (Mathisen et al., 2022; Rosset et al., 2022; Washington-Simon, 2024; Williamon et al., 2006), and one (5%) had all four components (Perkins et al., 2017). The following sections report how our included studies conceptualized each of the four mental health literacy components.

#### Recognition of mental health disorders

The recognition of mental health disorders was reflected in nine studies through either educating artists on mental health disorder symptoms (Cardinal and Hilsendager, 1997; Clements-Cortés et al., 2024; Mathisen et al., 2022; Shaw et al., 2020; Smith, 2021) or developing self-awareness (Harrison et al., 2019; Perkins et al., 2017; Qu, 2022; Rosset et al., 2022). There was one qualitative, three experimental, one mixed method, two cross-sectional, and two case studies. Cardinal and Hilsendager (1997) defined the ten curricular components of their dancer wellness education model, which included “psychology.” Under this curriculum component, they emphasized the importance of recognizing early warning signs of mental health disorders to facilitate appropriate access to services. The cross-sectional study by Cardinal et al. (2020) examined the dancer wellness education provided by post-secondary institutions. “Psychology” was identified as one of the possible course offerings in dance wellness education. The case study conducted by Shaw et al. (2020) discovered that a music teacher without psychotherapy training successfully identified a music student with problematic levels of MPA by identifying common MPA symptoms. The authors discussed the potential advantages of training music teachers to recognize symptoms of mental health issues in their students and the importance of self-awareness, which requires the individual to be attuned to their mental state. This may facilitate the recognition of mental health disorders.

Clements-Cortés et al. (2024) provided music psychotherapy sessions designed to cover the recognition of MPA symptoms. Participants of this study reported having a refreshed recognition of MPA symptoms and more self-awareness and adaptive cognitions about MPA. Mathisen et al. (2022) acknowledged that improving mental health literacy included raising awareness about mental health disorders and symptoms. These authors included understanding “physical and mental health (definition, understanding, symptoms, frequency)” (p. 4) as part of an intervention designed to increase mental health literacy in dancers. Finally, Žilinskas and Lesinskienė (2023) found that arts students ranked second highest in suicide literacy, following biomedical students, in higher education settings in Lithuania. Overall, most studies included the recognition of symptoms of mental health issues more generally instead of mental health disorder-specific symptoms.

#### Knowledge of risk factors and causes and use of mental health information and support services

Fourteen studies discussed the knowledge of risk factors and uptake of mental health information and use of support services (Atkins, 2009; Barnett et al., 2019; Cardinal et al., 2020; Ginsborg et al., 2012; Kaufman et al., 1996; Mathisen et al., 2022; Rosset et al., 2022; Perkins et al., 2017; Stuckey et al., 2021; Torres-McGehee et al., 2011; Walker and Nordin-Bates, 2010; Washington-Simon, 2024; Williamon et al., 2006).

Five studies were intervention studies (Kaufman et al., 1996; Mathisen et al., 2022; Rosset et al., 2022; Stuckey et al., 2021; Torres-McGehee et al., 2011), and were or are being conducted in school or university settings. Eight studies were descriptive (Barnett et al., 2019; Cardinal et al., 2020; Ginsborg et al., 2012; Perkins et al., 2017; Walker and Nordin-Bates, 2010; Williamon et al., 2006; Washington-Simon, 2024; Uriegas et al., 2024). Of these, five sampled student populations. Atkins’ (2009) case studies on seven conservatoires in Britain found that conservatoires conceptualized provision of support services as offering psychological services such as counseling as well as accessibility to information, support, funding, and treatment. Williamon et al. (2006) highlighted a need for more curriculum initiatives aiming to increase music students’ knowledge of mental health after finding that students primarily sought advice from their instrument teachers rather than healthcare professionals. Conservatory students reportedly had little awareness of existing support services and mental health risk factors (Perkins et al., 2017). It is possible that the psychology courses by Cardinal et al. (2020) could include knowledge of risk factors and causes related to mental health literacy, but this was not explicit. Finally, a cross-sectional study surveying gender diverse marching band musicians in US collegiate institutes found that almost all (96%) previously identified a mental health condition, and most of them (79.5%) sought mental health support (Uriegas et al., 2024).

Four studies focused on or included professional populations. An exploratory study by Walker and Nordin-Bates (2010) found that dancers in one elite dance company primarily relied on self-learned strategies when dealing with mental health challenges. Although not stated in the article, this could imply a lack of use of professional services. Barnett et al. (2019) found that participating in an exhibition designed to reduce stigma related to mental health within the general community increased knowledge of mental health issues and services in the exhibiting visual artists themselves. The literature review by Ginsborg et al. (2012) highlighted the need for musicians, including professionals, to be aware of personal risk factors and the underlying causes of various physical and mental health problems, such as music performance anxiety. Finally, Washington-Simon’s (2024) dissertation highlighted that Hip Hop musicians perceived that financial instability, differential treatment from society (because of stereotypes specific to Hip Hop community about being preoccupied with drugs, violence and sex), are risk factors to mental health disorders.

#### Attitudes that promote recognition and help-seeking

Ten studies investigated attitudes related to mental health, most of which studied musicians. Of these studies, three investigated the attitudes held by musicians towards mental health (Ginsborg et al., 2012; Perkins et al., 2017; Rosset et al., 2022), one discussed the importance of attitude in promoting healthy behaviors (Barton and Feinberg, 2008), one delivered an intervention that included fostering positive perspectives during adversity (Bartos et al., 2022a, 2022b), and one administered a music psychotherapy intervention in a group setting, which allowed participants to foster a sense of connection and support with their peers, allowing them to discover that they can reach out to others as a tool for helping them cope with MPA (Clements-Cortés et al., 2024). A study evaluating psychological help seeking attitudes among higher education students in Lithuania, which included arts students, but the report did not provide any subgroup analyses for arts students (Žilinskas and Lesinskienė, 2023).

In their literature review, Ginsborg et al. (2012) noted that professional musicians were either unable or not motivated to prioritize physical and mental wellness despite high levels of health issues within the profession. Music students tended to place greater importance in mental health, as demonstrated by Perkins et al. (2017) and Rosset et al. (2022). Rosset et al. (2022) also found that music students relied primarily on social support as a coping strategy in stressful situations. Marching band musicians were reported to be open to seeking mental health support, which positively correlated with their favorable attitudes toward mental healthcare (Moore et al., 2024). Furthermore, the authors noted that participants did not frequently cite a lack of knowledge about available services or the belief that no one would understand their mental health problems as significant barriers to seeking support (Moore et al., 2024). However, a gender-diverse subgroup of marching band artists from the same study (Uriegas et al., 2024) reported slightly less positive attitudes toward seeking mental health support, expressing concern that “no one will understand their problems.” Hip Hop musicians expressed that the perception of being undervalued coupled with financial barriers limited their options to mental healthcare accessibility (Washington-Simon, 2024).

Although perspective towards health was not measured in this study, Barton and Feinberg (2008) reflected after completion of a study designed to promote healthy behaviors in music students that future health promotion strategies should target changing attitudes towards health and well-being. Bartos et al. (2022a, 2022b) employed a yoga and mindfulness intervention that focused on cultivating positive perspectives towards adversity, which resulted in enhanced empowerment and positive attitudes towards help-seeking in music students. Finally, one of the studies (Study 1) in Griffith’s (2023) dissertation reported that visual artists used art to process emotions, helping them better understand and recognize their mental health challenges. Art was also seen as a way to “open the door” for communicating about mental health conditions and reaching out for help—particularly through digital platforms like Instagram, where artists described the benefits of building an online support system that encouraged help-seeking for themselves and for others.

#### Understanding the concept of stigma

Seven studies conceptualized mental health stigma within the artistic environment, four of which were in music, one in visual arts, and one in dance. Four of these articles reported that music students tend to not discuss mental health issues (Mathisen et al., 2022; Perkins et al., 2017; Smith, 2021; Williamon et al., 2006); however, support from instrumental teachers and from the musical community emerged as facilitators of the mental and physical well-being of students. The fifth study, Shaw et al. (2020), identified that there is stigma around working with healthcare professionals and found that one possible solution may be to train music teachers to offer mental skills training. Although stigma was mentioned in the four articles above, it was not the core focus of any of them. Mathisen et al. (2022) acknowledged that culture- and self-stigma contribute to low mental health literacy among dancers. Smith (2021) suggested that concealment of mental health challenges within the music industry may be due to fear of negative judgment or related to employment prospects. Among Hip Hop musicians, stigma had a substantial impact on mental health attitudes and hindered their willingness to seek support. They also expressed concern that potential judgment from fans, peers, and industry partners often limited their ability to prioritize their own mental health (Washington-Simon, 2024).

### Operationalizing mental health literacy

**Figure 1:**
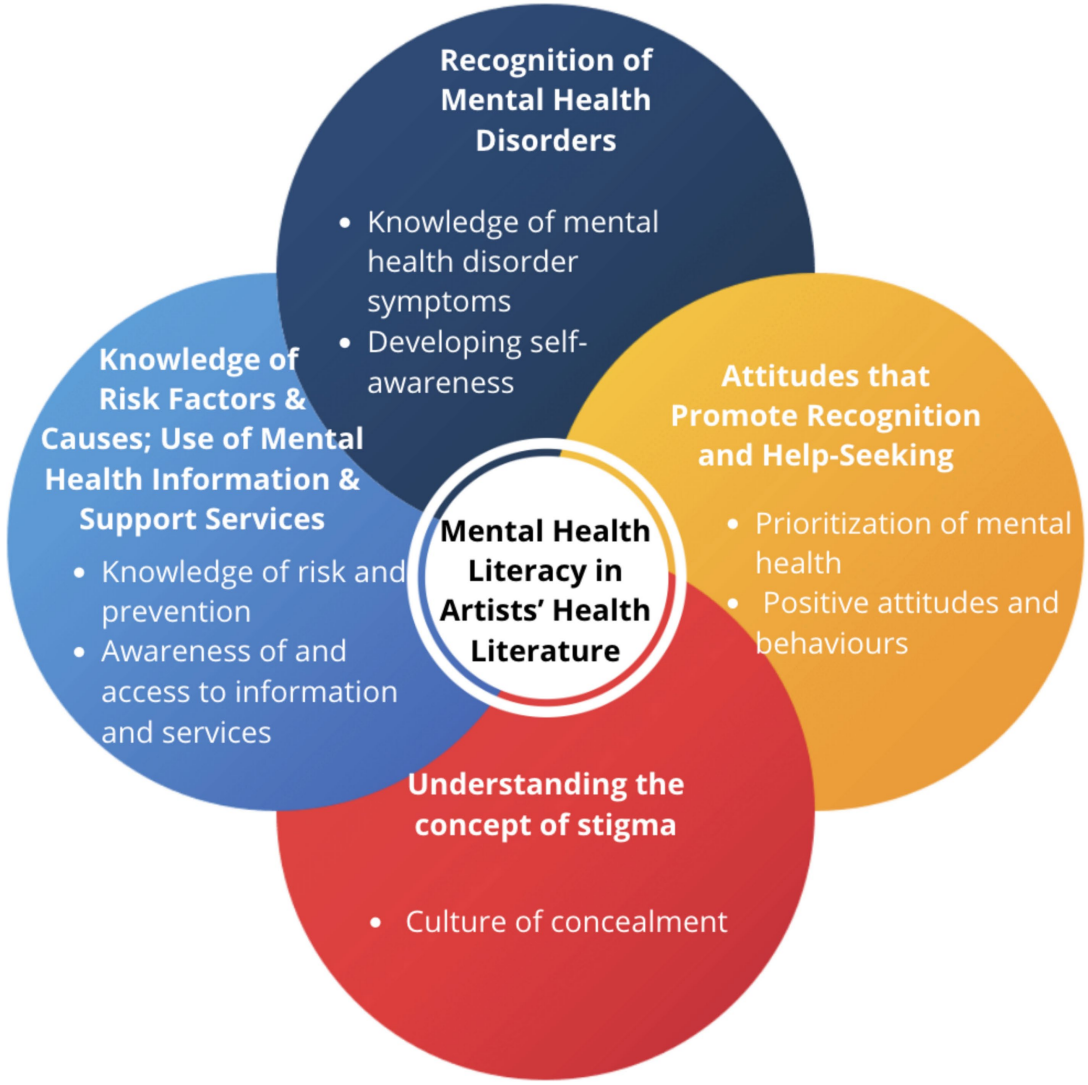
Four components of mental health literacy (Jorm, 2000) operationalized in artists’ health literature.

We summarize how the authors of the included studies operationalized the four components of mental health literacy (Figure 1). All studies in our review operationalized at least one component of mental health literacy. In these studies, the investigators operationalized these components through interventions (such as mobile applications), assessments (using validated instruments or qualitative assessments that target at least one aspect of mental health literacy), and educational sessions (courses). Table 2 presents the categorized operationalizations for each component of mental health literacy along with relevant, illustrative excerpts from the respective studies.

**Table 2:** Operationalization of mental health literacy in included studies.

Components and categories	Description	Illustrative quotes from included studies
Component: Recognition of mental health disorders	This component pertains to knowledge of symptoms of mental health conditions and developing self-awareness. Self-awareness is included under this component because artists can identify symptoms in themselves using self-awareness.	
Category: Knowledge of mental health disorder symptoms	This category focuses on knowledge of the symptoms of various mental health conditions. Information in studies that included the recognition of symptoms of mental health challenges (not specific to disorders) is placed under this category.	“Artists discussed how the Rural Art Roadshow allowed them to increase their knowledge of mental health issues and services. This occurred by artists engaging with service providers that attended the Rural Art Roadshow and others who also had a mental health illness, or were caring for someone with mental health challenges: ‘Just knowing about the art show actually helped me to learn more about my illness and develop skills around managing it. But I’ve also been in the opportunity where I’ve been able to gain knowledge and information (Participant 1)’” (Barnett et al., 2019, p. 6)
Category: Developing self-awareness	This category consists of developing awareness of the individual’s mental state, which includes their thoughts and emotions. The development of self-awareness was often not specifically for the purpose of identifying mental health disorder symptoms. However, if combined with the knowledge of mental health disorder symptoms, self-awareness could be used by artists to recognize mental health disorder symptoms in themselves.	“It can help individuals understand their own mental health from the aspects of emotion, thinking, consciousness, and living habits.” (Qu, 2022)
Component: Knowledge of risk factors and causes and use of mental health information and support services	This component contains two categories: provision of preventive information or coping strategies, rather than symptom identification, and ability to find and use mental health services.	
Category: Knowledge of risk and prevention	This category focuses on the knowledge of risk factors of various mental health issues as well as preventive actions artists can take. Some interventions focused specifically on preventing specific conditions, while others aimed towards overall well-being. Topics such as nutrition and stress management strategies were addressed by interventions targeting the development of knowledge in this area.	“The questionnaire further asked about the perceived level of knowledge about health risks for musicians… and about health protective measures for musicians.” (Rosset et al., 2022, p. 5)
Category: Awareness of and access to information and services	This category encompasses the availability as well as the ability to access and select appropriate support services. Availability of services constitutes literature that discusses support services offered by institutions. The uptake of services and the ability to identify when one should use a support service is included under this subtheme.	“The aim of the first part of the programme is to raise students’ consciousness of their own health by providing them with a basic theoretical knowledge of the physiological processes that underlie music performance including movement and breathing, and the neurological basis of learning. They are also introduced to some selected aspects of applied psychology, learning, for example, how relaxation procedures and psychotherapy can be used for coping with stress.” (Ginsborg et al., 2012, p. 360) “It may be that this apparent reliance on instrumental teachers for advice and guidance is symptomatic of a general deficiency of information – reflecting, in other words, not a positive decision but one reached through lack of knowledge of the alternatives.” (Williamon et al., 2006, p. 425)
Component: Attitudes that Promote Recognition and Help-Seeking	This component constitutes two categories: the prioritization of mental health and changing attitudes towards help-seeking.	
Category: Prioritization of mental health	This category focuses on the level of importance artists ascribe to mental health. Prioritization of time, financial, and cognitive resources in relation to mental health were discussed.	“On average, the respondents reported to be rather health conscious (M = 3.98) and ascribed high importance to health overall (M = 4.45) and especially to health for musicians (M = 4.68).” (Rosset et al., 2022, p. 7) “Financial barriers to mental health support highlight the financial constraints and limited access to comprehensive health insurance faced by Hip Hop artists, underscoring the financial barriers to mental health support within the industry.” (Washington-Simon, 2024, p. 70)
Category: Positive attitudes and behaviors	This category focuses on changing one’s attitude towards both mental health symptoms and health-promoting behaviors. Three types of changes in attitude are covered within this category: cultivating non-judgemental attitudes, maintaining positive perspectives, and fostering empowerment to pursue health-promoting behaviors.	“It is important for music educators and health professionals to understand that college students beginning an intensive course of music study often have generalized anxiety and insecurity that might be reduced if they are encouraged to have a positive attitude towards health behaviors.” (Barton and Feinberg, 2008, p. 52) “In discussing these results, we postulated that the program’s focus on invoking a sense of agency and self-responsibility—drawing from both yoga and mindfulness—might have raised students’ consciousness to self-engage in meaningful actions for independently taking care of their own health.” (Bartos et al., 2022a, 2022b, p. 16)
Component: Understanding the concept of stigma	This theme refers to the perception of mental health disorders within performing arts communities.	
Category: Culture of concealment	This category focuses on the culture of hiding ailments and lack of disclosure of mental health challenges within the performing arts. The category also includes issues such as fear of vulnerability and fear of judgement in addition to the internalization of societal these mechanisms.	“One participant reported that they had at times not spoken about their mental health because they felt ashamed of their mental illness, however, the Rural Art Roadshow provided them with an opportunity to speak out and feel empowered: ‘It’s something that has given me an opportunity to talk in public. There’s been a lot of secrecy around my mental illness. My family of origin are still in denial about it so for me to be able to speak out about it has been really empowering. So through the Roadshow I feel I’ve been given a voice and been able to talk about issues around mental illness. (Participant 1)’” (Barnett et al., 2019) “Secondly, students reported that health and wellbeing is something discussed infrequently at the conservatoire (*n* = 2, 10%) or that people choose not to disclose (*n* = 4, 20%)” (Perkins et al., 2017, p. 9) “You can face some sort of a stigma or you can face some sort of misunderstanding… when you are truly feeling horrible, but people can just ask you, ‘Why cannot you just…feel normal?’ Or, ‘Just do not worry’…people just do not understand that it does not work that way unfortunately.” (Griffith, 2023, p. 32)

#### Recognition of mental health disorders

Seven studies operationalized the first component of mental health literacy. Harrison et al. (2019) investigated the impacts of a mobile application that monitors various well-being components of physical and psychological well-being. Although this study did not specifically target mental health disorders, the psychological components monitored by the application included stress, fatigue, and sleep quality, which can be symptoms of mental health disorders depending on the frequency and intensity at which they are experienced. Perkins et al. (2017) found that 95% of university music students and recent graduates reported high levels of self-awareness; however, this referred to an overall mental state and was not specific to the recognition of mental health disorders. An experiment by Rosset et al. (2022) included self-awareness as part of a general health course for musicians. The course itself contained mental health components, but it was not specified as to whether self-awareness was for physical or mental health purposes. Shaw et al. (2020) conducted a study examining whether students with MPA would achieve similar results when receiving acceptance and commitment coaching (ACC) from a singing teacher compared to receiving it from a clinical psychologist. As self-awareness was integrated into the ACC, we decided to include this component as representative of operationalizing mental health literacy. Qu (2022) found that after participating in an expressive art therapy intervention, post-modern novelists developed a greater sense of self-awareness. Clements-Cortés et al. (2024) incorporated recognizing performance anxiety symptoms in an intervention for musicians. Barnett et al.’s (2019) Rural Art Show allowed creative artists to increase their knowledge about mental health issues, symptoms and services.

#### Knowledge of risk factors and causes and use of mental health information and support services

Seven studies operationalized the second component of mental health literacy. Kaufman et al. (1996) examined the impact of an intervention targeting dieting behaviors and stress fractures on adolescent ballet students. The intervention consisted of lectures on medical, nutritional, and psychological issues, as well as additional counseling and physical therapy support. The lectures provided information on the causes of eating disorders, which are classified as a mental health disorder under the Diagnostic and Statistical Manual of Mental Disorders, 5th Edition (2013). Mathisen et al.’s (2022) intervention aimed to increase mental health literacy by teaching university dance students about nutrition, eating disorders, and recovery strategies. The intervention by Torres-McGehee et al. (2011) focused entirely on the prevention and consequences of eating disorders by providing information on nutrition, exercise, and depression to collegiate dance students.

Three studies examined populations outside of dance. Rosset et al. (2022) examined the impacts of a musicians’ health course on post-secondary music students. The authors observed moderate baseline health knowledge among music students, with the intervention group showing increased knowledge post-test. The intervention encompassed general health and stress management strategies, but it was unclear whether the intervention had any focus on mental health risks. Stuckey et al. (2021) is conducting an ongoing longitudinal study on the impacts of a holistic, resilience-focused intervention program on the mental well-being, creativity, and lifestyle habits of college circus students. The intervention targets physical literacy and is based on the Circus for Development Model. This model encapsulates psychology as a component of physical literacy. This particular intervention will include teaching participants about community resources that are available, but the article does not specify whether these community resources are mental health-specific or more relevant to general health. Guptill et al. (2022) investigated the validity and reliability of the MHL-Q19, which is a tool that assesses general health literacy, in musicians. The tool assesses the individual’s ability to access, understand, appraise, and apply information related to performance health. Participants in this study were post-secondary music students. Even though the MHL-Q19 did not include specific components of mental health literacy, three of the questions may be adapted to understand mental health literacy, including finding risks about performance health, understanding risks related to performance health, and finding reliable information on performance health. Griffith (2023) interviewed visual artists about mental health and social media. Lack of information and ability to navigate health systems were identified as two barriers to mental health treatment in artists. Advocacy and mental health awareness were identified as two protective factors.

#### Attitudes that promote recognition and help-seeking

Six studies operationalized the third component of mental health literacy. Barton and Feinberg (2008) investigated the impacts of an educational intervention targeting health promotion and injury prevention; however, it is unclear whether these interventions are specifically targeting mental health promotion. Bartos et al. (2022a, 2022b) investigated the impacts of a multi-faceted yoga and mindfulness intervention designed to enhance well-being during the COVID-19 pandemic. The intervention aimed to foster positive perspectives towards adversity by developing non-judgmental attitudes toward emotional and cognitive experiences. After qualitative analysis, the authors found the intervention led to an enhanced sense of empowerment in participants regarding their mental health, which they attributed to the multi-faceted nature of the program. Rosset et al. (2022) assessed the importance of mental health and perceived health consciousness to music students through questionnaires. Washington-Simon (2024) found that hip hop artists juggle competing work and financial demands that impede the prioritization of mental health. Similarly, Uriegas et al. (2024) identified lack of time as the primary barrier to marching band artists receiving professional mental health services. This was supported by Moore et al.’s (2024) findings where marching band artists had varied perceptions of the necessity of professional help for mental health services.

#### Stigma

Stigma was operationalized in four studies. Uriegas et al. (2024) identified that marching band artists are fearful of directors knowing about their mental health issues. Similarly, Washington-Simon (2024) found that fear of vulnerability occurs as artists face expectations and judgement from colleagues and fans. Griffith (2023) discussed how artists may internalize these judgements made by others and develop self-stigma. Three articles also alluded to a culture of concealment of mental health challenges in fear of receiving judgment or negative biases from others (Mathisen et al., 2022; Smith, 2021; Williamon et al., 2006). Despite this, Moore et al. (2024) found that stigma was not a significant barrier to help-seeking in marching band artists.

### Measuring mental health literacy

We extracted how mental health literacy was measured using two categories and eight subcategories: type of measurement (survey, scaling, qualitative, and observational), and level of measurement (nominal, ordinal, ratio, and open-ended).

Table 3 displays the type of study (descriptive, intervention, literature review, instrument development) and the measure that was utilized, organized by mental health literacy component for each included study. Among the 18 studies incorporating a quantitative component (4 with mixed methods), none measured all four components of mental health literacy. Component Two, focused on Knowledge of Risk Factors and Causes; Use of Support Services, was the most frequently measured (*n* = 9; 50%). More than half (*n* = 11; 61.1%) of the 18 studies employed an ordinal level of measurement, six used a nominal scale, and one included open-ended questions in their questionnaire. Additionally, one study used both an ordinal and a ratio level of measurement. Out of the 26 studies identified in this review, eight explored various components of mental health literacy through individual interviews and one used a focus group. Meanwhile, two studies consisted of literature reviews and consequently did not measure any component of mental health literacy in artists.

**Table 3:** Type of study and measure organized by mental health literacy component.

Article	Type of study and its description	Recognition of mental health disorders	Knowledge of risk factors and causes; use of support services	Attitudes that promote recognition and help-seeking	Understanding the concept of stigma
Atkins (2009)	Descriptive study. Case studies on uptake and perception		Self-report questionnaire (nominal)		
Barnett et al. (2019)	Descriptive study. Data on experiences of artists participating in an exhibition.		Individual interviews		
Barton and Feinberg (2008)	Music and health course on health promotion and prevention strategies. 8 sessions over 8 weeks given by researcher		Assessment of baseline vs. acquired knowledge (ratio) Self-report questionnaire (ordinal)	Assessment of baseline vs. acquired knowledge (ratio)	
Bartos et al. (2022a, 2022b)	CRAFT Intervention consisting of yoga, mindfulness, emotional intelligence, and positive psychology. 23 weeks, 1 h per week + 2 h of weekly practice. Course delivered in-person and virtually.			Self-report questionnaire of open-ended questions (descriptive)	
Cardinal and Hilsendager (1997)	Literature review followed by a descriptive study	Individual interviews			
Cardinal et al. (2020)	Descriptive study.		Self-report questionnaire (nominal)		
Clements-Cortés et al. (2024)	Group music psychotherapy offered for six weeks in-person or via online.	Individual interviews and self-report questionnaire (nominal).		Individual interviews.	
Ginsborg et al. (2012)	Literature review.		Data synthesis	Data synthesis	
Griffith (2023)—Study 1	Descriptive study.			Individual interviews	
Guptill et al. (2022)	Validation study.		Self-report questionnaire (ordinal)		
Harrison et al. (2019)	Wellness monitoring mobile application.	Self-report questionnaire(nominal) and focus group interviews			
Kaufman et al. (1996)	Lectures (lasting 12 months) given by a physical therapist, an endocrinologist, a nutritionist, and a clinical psychologist.		Self-report questionnaire (nominal)		
Mathisen et al. (2022)	Physical and mental health (definition and symptoms), nutrition, recovery strategies, and impacts on performance Monthly 90-min workshops over 3 months	Self-report questionnaire (ordinal)	Self-report questionnaire (ordinal)	Self-report questionnaire (ordinal)	
Moore et al. (2024)	Cross-sectional study		Self-report questionnaire (ordinal)	Self-report questionnaire (ordinal)	
Perkins et al. (2017)	Descriptive study.	Individual interviews	Individual interviews	Individual interviews	Individual interviews
Qu (2022)	30-day reception music therapy intervention.	Self-report questionnaire (ordinal)			
Rosset et al. (2022)	Courses on musicians’ health for two semesters.	Self-report questionnaire (ordinal)	Self-report questionnaire (ordinal)	Self-report questionnaire (ordinal)	
Shaw et al. (2020)	Acceptance and commitment intervention for six, 60-min sessions over 3 months.	Self-report questionnaire (ordinal)			Self-report questionnaire (ordinal)
Stuckey et al. (2021)	1-year. Intervention consisting of group webinars, small group information sessions, one-on-one Zoom meetings, and sending external resources.		Self-report questionnaire (ordinal)		
Torres-McGehee et al. (2011)	Intervention consisting of an education program to prevent eating disorders. Eight, 45-min sessions over 8 weeks (2 sessions per week) led by peers.		Self-report questionnaire (nominal)		
Walker and Nordin-Bates (2010)	Descriptive study.		Individual interviews		
Washington-Simon, 2024	Descriptive study.		Individual interviews	Individual interviews	Individual interviews
Williamon et al. (2006)	Introductory seminar on music and health.	Self-report questionnaire (ordinal)	Self-report questionnaire (ordinal)	Self-report questionnaire (ordinal)	
Smith (2021)	Literature review of existing programs in post-secondary institutions.		Data synthesis		
Uriegas et al. (2024)	Cross-sectional study.	Self-report questionnaire (nominal)		Self-report questionnaire (ordinal)	
Žilinskas and Lesinskienė (2023)	Cross-sectional study.	Self-report questionnaire (ordinal)		Self-report questionnaire (ordinal)	

The questionnaires administered in studies that measured mental health literacy components varied considerably, many of which were developed by the study investigators and were not validated instruments. Eleven studies utilized measures to examine the impact of mental health literacy related interventions on various outcomes for participants. For instance, Rosset et al. (2022) measured three of four mental health literacy components after administering the courses on musician’s health over two semesters. Harrison et al. (2019) assessed users’ level of recognition of mental health disorders after using the wellness monitoring mobile application. Guptill et al. (2022) developed a validated instrument for measuring musicians’ health literacy; however, it is worth noting that this instrument asked questions about performance health issues and therefore, does not make any explicit inclusion of any of the components of mental health literacy. The validated questionnaire that was most commonly administered was the Attitudes Toward Seeking Professional Psychological Help-Short Form Scale (Picco et al., 2016) which measures Component Three, Attitudes that Promote Recognition and Help-Seeking (see Table 1 for list of validated questionnaires administered in studies).

## Discussion

This scoping review aimed to identify and summarize how mental health literacy is conceptualized, operationalized, and measured in the performing and creative arts literature. Surprisingly, only one included study explored all four components of mental health literacy, and just one study provided a definition of this concept. These findings suggest that components of mental health literacy are not commonly investigated in the artists’ health literature, despite the fact that mental health literacy was defined over two decades ago (Jorm, 2000), and that mental health literacy assessments and interventions have been established in other performance-related disciplines, such as sports medicine (Bu et al., 2020; Vella et al., 2021; Wang et al., 2022). In elite sports, seven consensus statements from prominent sports psychiatry and psychology associations have all declared that increasing mental health literacy is one of the primary strategies for preventing and treating mental health disorders and symptoms, as well as preventing stigma, in elite athletes (Gorczynski et al., 2021).

The limited exploration of all components of mental health literacy in the included studies raises concerns about the perpetuation of stigma around mental health in the artist community. Mental health stigma is recognized as a multidimensional process of objectifying and dehumanizing an individual or self who is known to have or appears to have a mental disorder (Corrigan and Watson, 2004; Pescosolido and Martin, 2015). Previous studies revealed that mental health stigma predicted a reduced level of help-seeking attitudes and behaviors (Clement et al., 2015; Mendoza et al., 2015; Vassallo et al., 2019). Mental health stigma is significantly inversely related to the recognition of need for psychotherapeutic help, stigma tolerance, and interpersonal openness (Mendoza et al., 2015). Interestingly, mental health stigma was also the least explored in all of the included studies. The concept of stigma within the artistic environment was almost exclusively examined among musicians, which reported that musicians tend to conceal medical concerns, with limited open discussions about health and well-being. Our findings highlight the need for further research to examine stigma within all artistic disciplines and develop strategies to combat it effectively. By addressing stigmatizing attitudes and misconceptions of mental health through interventions based on a comprehensive understanding of mental health literacy, institutions can encourage help-seeking attitudes of artists so that they can access support services without fear of judgment or discrimination.

In our review, over half of the included studies investigated only one of the four components of mental health literacy, suggesting a lack of comprehensive awareness of all mental health literacy components in the artists’ health literature. Our findings are consistent with the current state of the literature on mental health literacy in all disciplines, indicating that studies often do not simultaneously address all facets of this concept (Kutcher et al., 2016). The second component of mental health literacy, Knowledge of Risk Factors and Causes, and the Use of Mental Health Information and Support Services, was explored in a larger number (*n* = 14) of studies. Studies on musicians underscored the importance of understanding risk factors, with interventions and courses addressing musicians’ knowledge of mental health risks and protective measures. However, it was noted that the type and degree of support services offered to music students varied across institutions, indicating a need for more standardized and comprehensive support. In non-musician artists, several studies highlighted the importance of educating performing artists on mental health disorder symptoms and developing self-awareness. For example, interventions aimed at increasing mental health literacy in dancers included educating them about the symptoms of psychological disorders and understanding physical and mental health. Similarly, studies involving musicians emphasized the need for awareness of personal risk factors and causes of performance anxiety. These findings suggest that interventions facilitating recognition of mental health disorders can enhance the artists’ ability to identify and address potential mental health issues.

The implications of the findings from this review are far-reaching, spanning clinical practice, research, and community levels. For clinical practice, determining the level of mental health literacy in artists allows practitioners and arts educators to tailor educational programs that address specific areas of limited knowledge. As recommended by Barton and Feinberg (2008), health literacy promotion interventions should also include educational strategies designed to specifically affect attitudinal change with appropriate measurements used to measure mental literacy outcomes. Mental health literacy interventions should include all four components and be contextually developed and applied in the artists’ workplace settings. Kutcher et al. (2016) suggested that mental health literacy interventions must fit the context in which they are to be deployed. Therefore, tailored mental health interventions that consider the unique occupational contexts of each artistic discipline may prove more effective than a one-size-fits-all approach. It is possible, for example, that mental health literacy interventions for dancers may focus more heavily on recognizing the early signs of eating disorders and performance anxiety, both of which have been considered as prominent psychological disorders among dancers (Dwarika and Haraldsen, 2023), compared to creative writers, who tend to suffer from melancholic states and mood disorders (Andreasen, 2008).

Mental health literacy interventions have demonstrated their effectiveness in promoting resilience, reducing stigma, and increasing confidence in seeking mental health information and professional support across different population samples including athletes (Bu et al., 2020; Vella et al., 2021), medical students (Kurki et al., 2021), and school-aged children (Renwick et al., 2022). Performing arts education programs should incorporate mental health literacy education and training to support performers in maintaining their mental well-being. Research indicates that performing arts students often establish a trusting relationship with their teachers, and consult them (Mahony et al., 2022; Williamon et al., 2006). Therefore, one effective approach to introducing mental health literacy concepts among students may include enhancing the mental health literacy of teachers so that they can more proactively recognize early signs of mental health risk factors in their students. At the research level, a robust knowledge base on mental health literacy in artists would allow researchers to gain a deeper understanding of artists’ mental health literacy and identify effective interventions to support artists’ mental health. To advance our understanding in mental health literacy in artists, there is a pressing need for a standardized definition and measurement tool that could facilitate more robust research investigations. Future research could also explore the specific relationship between mental health literacy and common mental health challenges faced by performing artists, such as performance anxiety, burnout, and depression. By unraveling these connections, tailored interventions and support mechanisms can be developed to promote mental health literacy. Furthermore, longitudinal studies could be conducted to track changes in mental health literacy among artists over time, examining how factors such as training, experience, and exposure to different artistic environments impact mental health literacy development. Comparative studies across different artistic disciplines and cultural contexts may also add valuable insights into the universality of mental health literacy concepts and the effectiveness of interventions across diverse artistic disciplines.

Finally, collaborative efforts between researchers, educators, mental health professionals, and arts organizations can open dialogue and create synergies on designing and implementing comprehensive mental health literacy interventions tailored for different artistic professions. This scoping review has several limitations. The inclusion criteria restricted the review to English language publications, which may have excluded relevant studies published in other languages. Although the researchers attempted to be as comprehensive as possible of all artistic disciplines during the search process, due to the vast array of artistic disciplines, it is possible that articles focusing on extremely niche disciplines may not have been found during the search process. In addition, due to the imbalance in research that has been conducted across the artistic disciplines, the conceptualization of mental health literacy may evolve as research develops in artistic disciplines outside of dance and music.

A key limitation of the included literature was the lack of clear and consistent definitions of mental health literacy. Many studies did not define the term or failed to incorporate all four core components of the construct—recognition of disorders, knowledge of support and risk factors, attitudes promoting help-seeking, and understanding of stigma. This inconsistency introduces subjectivity in interpretation and limits the comparability and generalizability of findings across studies. Furthermore, the absence of validated tools for measuring mental health literacy among performing and creative artists represents a major gap in the field, which discourages the ability to measure mental health literacy or evaluate education interventions.

In alignment with scoping review methodology, we did not conduct a formal appraisal of the methodological quality of included studies. This limits our ability to comment on the strength of the evidence base, as some findings may be drawn from lower-quality or exploratory research. In addition, our review can be at risk of publication bias, as we relied on peer-reviewed literature, potentially excluding unpublished or non-traditional forms of scholarship that may be particularly relevant in artistic contexts. Thematic synthesis also involves a degree of subjective interpretation, especially when reviewing studies that lack conceptual clarity.

Despite these limitations, the review provides a valuable foundation by mapping the current state of mental health literacy research in performing and creative artists and highlighting important gaps in the literature. These include the need for (1) more consistent use of mental health literacy; (2) greater representation of diverse artistic disciplines and contexts; (3) development and validation of context-specific mental health literacy measurement tools; and (4) more robust study designs that allow for assessment of outcomes and intervention effectiveness. Addressing these gaps is essential to advancing the field and promoting mental health literacy in artist populations in meaningful and equitable ways.

Our scoping review underscores the importance of advancing research and practice in mental health literacy in artists. By addressing the gaps in conceptualization, operationalization, and measurement of this construct, researchers and practitioners can develop targeted interventions, reduce stigma, improve treatment outcomes, and foster a supportive environment that recognizes and prioritizes mental health in the context of artistic expression and creativity.

## References

[ref1] Alpheis S. Altenmüller E. (2024). Comparison of perfectionism between music and medical students and its association with anxiety. Med. Probl. Perform. Art. 39, 82–92. doi: 10.21091/mppa.2024.2011, PMID: 38814127

[ref2] Anderson L. M. (2011). Myself or someone like me: a review of the literature on the psychological well-being of child actors. Med. Probl. Perform. Art. 26, 146–149. doi: 10.21091/mppa.2011.3023, PMID: 21987069

[ref3] Andreasen N. C. (2008). The relationship between creativity and mood disorders. Dialogues Clin. Neurosci. 10, 251–255. doi: 10.31887/DCNS.2008.10.2/ncandreasen, PMID: 18689294 PMC3181877

[ref4] Atkins L. (2009). Health and wellbeing education in British conservatoires. In *Proceedings of the international symposium on performance science 2009* (pp. 219–223). European Association of Conservatoires.

[ref5] Barnett T. de Deuge J. Bridgman H. (2019). Promoting mental health through a rural art roadshow: perspectives of participating artists. Int. J. Ment. Heal. Syst. 13:44. doi: 10.1186/s13033-019-0302-y, PMID: 31249612 PMC6585087

[ref6] Barton R. Feinberg J. R. (2008). Effectiveness of an educational program in health promotion and injury prevention for freshman music majors. Med. Probl. Perform. Art. 23, 47–53. doi: 10.21091/mppa.2008.2010

[ref7] Bartos L. J. Funes M. J. Ouellet M. Posadas M. P. Immink M. A. Krägeloh C. (2022a). A feasibility study of a program integrating mindfulness, yoga, positive psychology, and emotional intelligence in tertiary-level student musicians. Mindfulness 13, 2507–2528. doi: 10.1007/s12671-022-01976-7

[ref8] Bartos L. J. Posadas M. P. Krägeloh C. (2022b). Perceived benefits of a remote yoga and mindfulness program for student musicians during COVID-19. Humanist. Psychol. 51, 303–328. doi: 10.1037/hum0000277, PMID: 40526774

[ref9] Bennett H. Allitt B. Hanna F. (2023). A perspective on mental health literacy and mental health issues among Australian youth: cultural, social, and environmental evidence. Front. Public Health 11:1065784. doi: 10.3389/fpubh.2023.1065784, PMID: 36741953 PMC9891461

[ref11] Breslin G. Haughey T. O’Brien W. Caulfield L. Robertson A. Lawlor M. (2018). Increasing athlete knowledge of mental health and intentions to seek help: the state of mind Ireland (SOMI) pilot program. J. Clin. Sport Psychol. 12, 39–56. doi: 10.1123/jcsp.2016-0039

[ref13] Bu D. Chung P. K. Zhang C. Q. Liu J. Wang X. (2020). Mental health literacy intervention on help-seeking in athletes: a systematic review. Int. J. Environ. Res. Public Health 17:7263. doi: 10.3390/ijerph17197263, PMID: 33020448 PMC7579198

[ref14] Cardinal M. K. Hilsendager S. A. (1997). A curricular model for dance wellness education in higher education dance programs. J. Dance Med. Sci. 1, 67–72. doi: 10.1177/1089313X9700100207

[ref15] Cardinal M. K. Rogers K. A. Cardinal B. J. (2020). Inclusion of dancer wellness education programs in US colleges and universities: a 20-year update. J. Dance Med. Sci. 24, 73–87. doi: 10.12678/1089-313X.24.2.73, PMID: 32456762

[ref16] Castaldelli-Maia J. M. Gallinaro J. G. Falcão R. S. Gouttebarge V. Hitchcock M. E. Hainline B.. (2019). Mental health symptoms and disorders in elite athletes: a systematic review on cultural influencers and barriers to athletes seeking treatment. Br. J. Sports Med. 53, 707–721. doi: 10.1136/bjsports-2019-100710, PMID: 31092400

[ref17] Clarke V. Braun V. (2013). Teaching thematic analysis: overcoming challenges and developing strategies for effective learning. Psychologist 26, 120–123.

[ref19] Clements-Cortés A. Pascoe H. Pranjić M. Nan F. (2024). An explanatory sequential pilot inquiry on music therapy and performance anxiety in university music education majors. Arts Psychother. 87:102114. doi: 10.1016/j.aip.2023.102114

[ref18] Clement S. Schauman O. Graham T. Maggioni F. Evans-Lacko S. Bezborodovs N.. (2015). What is the impact of mental health-related stigma on help-seeking? A systematic review of quantitative and qualitative studies. Psychol. Med. 45, 11–27. doi: 10.1017/S0033291714000129, PMID: 24569086

[ref20] Corrigan P. W. Watson A. C. (2004). At issue: stop the stigma: call mental illness a brain disease. Schizophr. Bull. 30, 477–479. doi: 10.1093/oxfordjournals.schbul.a007095, PMID: 15631240

[ref21] Dempsey E. Comeau G. (2019). Music performance anxiety and self-efficacy in young musicians: effects of gender and age. Music Perform. Res. 9, 60–79.

[ref22] Détári A. Egermann H. Bjerkeset O. Vaag J. (2020). Psychosocial work environment among musicians and in the general workforce in Norway. Front. Psychol. 11:1315. doi: 10.3389/fpsyg.2020.01315, PMID: 32676045 PMC7333236

[ref23] Duarte A. M. (2020). “Artists’ precarity in the context of their social integration,” in Precarious places: Social, cultural and economic aspects of uncertainty and anxiety in everyday life. Wiesbaden: Springer Fachmedien Wiesbaden, 19–39.

[ref24] Dwarika M. S. Haraldsen H. M. (2023). Mental health in dance: a scoping review. Front. Psychol. 14:1090645. doi: 10.3389/fpsyg.2023.1090645, PMID: 36968742 PMC10035338

[ref25] Ginsborg J. Spahn C. Williamon A. (2012). “Health promotion in higher music education,” in Music, health, and wellbeing, 356–366.

[ref26] Gorczynski P. Currie A. Gibson K. Gouttebarge V. Hainline B. Castaldelli-Maia J. M.. (2021). Developing mental health literacy and cultural competence in elite sport. J. Appl. Sport Psychol. 33, 387–401. doi: 10.1080/10413200.2020.1720045

[ref27] Griffith F. J. (2023). Can social media contact reduce stigma? Promoting empathy with the art and writing of people experiencing mental illness. (Doctoral Dissertation). Bowling Green State University.

[ref28] Gross S. Musgrave G. (2016). Can music make you sick? Music and depression a study into the incidence of musicians’ mental health part 1: Pilot survey report. Help musicians UK. [Internet]. Available online at: https://core.ac.uk/download/pdf/161104499.pdf.

[ref29] Guptill C. Slade T. Baadjou V. Roduta Roberts M. de Lisle R. Ginsborg J.. (2022). Validity and reliability of the musicians’ health literacy questionnaire, MHL-Q19. Front. Psychol. 13:886815. doi: 10.3389/fpsyg.2022.886815, PMID: 36211877 PMC9541534

[ref30] Harrison C. Ruddock-Hudson M. Ruddock S. Mayes S. O’Halloran P. Cook J. (2019). Wellness monitoring in professional ballet dancers: a pilot study. J. Sci. Med. Sport. 22, S86–S87. doi: 10.12678/1089-313X.061521b 33781371

[ref31] Jiao S. Slemon A. Guta A. Bungay V. (2022). Exploring the conceptualization, operationalization, implementation, and measurement of outreach in community settings with hard-to-reach and hidden populations: a scoping review. Soc. Sci. Med. 309:115232. doi: 10.1016/j.socscimed.2022.115232, PMID: 35964472

[ref32] Jorm A. F. (2000). Mental health literacy: public knowledge and beliefs about mental disorders. Br. J. Psychiatry 177, 396–401. doi: 10.1192/bjp.177.5.396, PMID: 11059991

[ref33] Jorm A. F. (2012). Mental health literacy: empowering the community to take action for better mental health. Am. Psychol. 67, 231–243. doi: 10.1037/a0025957, PMID: 22040221

[ref34] Kaleńska-Rodzaj J. (2020). Pre-performance emotions and music performance anxiety beliefs in young musicians. Res. Stud. Music Educ. 42, 77–93. doi: 10.1177/1321103X19830098

[ref35] Kaufman B. A. Warren M. P. Hamilton L. (1996). Intervention in an elite ballet school: an attempt at decreasing eating disorders and injury. Women's Stud. Int. Forum 19, 545–549.

[ref36] Kenny D. Driscoll T. Ackermann B. (2014). Psychological well-being in professional orchestral musicians in Australia: a descriptive population study. Psychol. Music 42, 210–232. doi: 10.1177/0305735612463950

[ref38] Kern A. Heininger W. Klueh E. Salazar S. Hansen B. Meyer T.. (2017). Athletes connected: results from a pilot project to address knowledge and attitudes about mental health among college student-athletes. J. Clin. Sport Psychol. 11, 324–336. doi: 10.1123/JCSP.2016-0028

[ref39] Keyes C. L. M. (2002). The mental health continuum: from languishing to flourishing in life. J. Health Soc. Behav. 43, 207–222. doi: 10.2307/3090197 12096700

[ref66] Khalil H. Peters M. D. Tricco A. C. Pollock D. Alexander L. McInerney P.. (2021). Conducting high quality scoping reviews-challenges and solutions. J. Clin. Epidemiol. 130, 156–160. 33122034 10.1016/j.jclinepi.2020.10.009

[ref40] King B. Berg L. Koenig J. Adair J. Tirado C. (2019). A revised occupational stress measure for popular musicians: pilot test of validity and reliability. Med. Probl. Perform. Art. 34, 85–91. doi: 10.21091/mppa.2019.2015, PMID: 31152650

[ref41] Kurki M. Gilbert S. Mishina K. Lempinen L. Luntamo T. Hinkka-Yli-Salomäki S.. (2021). Digital mental health literacy-program for the first-year medical students’ wellbeing: a one group quasi-experimental study. BMC Med. Educ. 21:1. doi: 10.1186/s12909-021-02990-4 34742258 PMC8571980

[ref42] Kutcher S. Wei Y. Coniglio C. (2016). Mental health literacy: past, present, and future. Can. J. Psychiatry 61, 154–158. doi: 10.1177/0706743715616609, PMID: 27254090 PMC4813415

[ref43] Kutcher S. Wei Y. McLuckie A. Bullock L. (2013). Educator mental health literacy: a programme evaluation of the teacher training education on the mental health & high school curriculum guide. Adv. Sch. Ment. Health Promot. 6, 83–93. doi: 10.1080/1754730X.2013.784615

[ref44] Kyaga S. Landén M. Boman M. Hultman C. M. Långström N. Lichtenstein P. (2013). Mental illness, suicide and creativity: 40-year prospective total population study. J. Psychiatr. Res. 47, 83–90. doi: 10.1016/j.jpsychires.2012.09.010, PMID: 23063328

[ref45] Kyaga S. Lichtenstein P. Boman M. Hultman C. Långström N. Landen M. (2011). Creativity and mental disorder: family study of 300 000 people with severe mental disorder. Br. J. Psychiatry 199, 373–379. doi: 10.1192/bjp.bp.110.085316, PMID: 21653945

[ref46] Lazarus R. S. Folkman S. (1984). Stress, appraisal, and coping. New York City: Springer Publishing Company.

[ref47] Li S. A. Stevens C. Zhang Ke Jiang C. (2024). Impact of the COVID-19 pandemic on Canadian performing and creative artists: an interpretive descriptive study using the social-ecological model. PLoS One 19:e0310369. doi: 10.1371/journal.pone.0310369, PMID: 39288119 PMC11407613

[ref48] Loveday C. Musgrave G. Gross S. A. (2023). Predicting anxiety, depression, and wellbeing in professional and nonprofessional musicians. Psychol. Music 51, 508–522. doi: 10.1177/03057356221096506

[ref49] Mahony S. E. Juncos D. G. Winter D. (2022). Acceptance and commitment coaching for music performance anxiety: piloting a 6-week group course with undergraduate dance and musical theatre students. Front. Psychol. 13:830230. doi: 10.3389/fpsyg.2022.830230, PMID: 35369260 PMC8972159

[ref50] Mathisen T. F. Sundgot-Borgen C. Anstensrud B. Sundgot-Borgen J. (2022). Intervention in professional dance students to increase mental health-and nutrition literacy: a controlled trial with follow up. Front. Sports Act. Living 4:727048. doi: 10.3389/fspor.2022.727048, PMID: 36213449 PMC9532567

[ref51] McGowan J. Sampson M. Salzwedel D. M. Cogo E. Foerster V. Lefebvre C. (2016). PRESS peer review of electronic search strategies: 2015 guideline statement. J. Clin. Epidemiol. 75, 40–46. doi: 10.1016/j.jclinepi.2016.01.021, PMID: 27005575

[ref52] Mcluckie A. Kutcher S. Wei Y. Weaver C. (2014). Sustained improvements in students’ mental health literacy with use of a mental health curriculum in Canadian schools. BMC Psychiatry 14, 1–6. doi: 10.1186/s12888-014-0379-4, PMID: 25551789 PMC4300054

[ref53] Mendoza H. Masuda A. Swartout K. M. (2015). Mental health stigma and self-concealment as predictors of help-seeking attitudes among Latina/o college students in the United States. Int. J. Adv. Couns. 37, 207–222. doi: 10.1007/s10447-015-9237-4

[ref54] Merritt R. K. Price J. R. Mollison J. Geddes J. R. (2007). A cluster randomized controlled trial to assess the effectiveness of an intervention to educate students about depression. Psychol. Med. 37, 363–372. doi: 10.1017/S0033291706009056, PMID: 17311685

[ref55] Moher D. Shamseer L. Clarke M. Ghersi D. Liberati A. Petticrew M.. (2015). Preferred reporting items for systematic review and meta-analysis protocols (PRISMA-P) 2015 statement. Syst. Rev. 4, 1–9. doi: 10.1186/2046-4053-4-1 25554246 PMC4320440

[ref56] Moore K. Uriegas N. A. Emerson D. M. Winkelmann Z. K. Harriell K. Torres-McGehee T. M. (2024). Barriers to and attitudes toward seeking mental health services among collegiate marching band artists. J. Athl. Train. 59, 506–513. doi: 10.4085/1062-6050-0368.23, PMID: 38243734 PMC11127682

[ref57] Munn Z. Peters M. D. Stern C. Tufanaru C. McArthur A. Aromataris E. (2018). Systematic review or scoping review? Guidance for authors when choosing between a systematic or scoping review approach. BMC Med. Res. Methodol. 18, 1–7. doi: 10.1186/s12874-018-0611-x 30453902 PMC6245623

[ref58] Osborne M. S. Kenny D. T. Holsomback R. (2005). Assessment of music performance anxiety in late childhood: a validation study of the music performance anxiety inventory for adolescents (MPAI-A). Int. J. Stress Manage. 12, 312–330. doi: 10.1037/1072-5245.12.4.312

[ref60] Patston T. Osborne M. S. (2016). The developmental features of music performance anxiety and perfectionism in school age music students. Perform. Enhanc. Health 4, 42–49. doi: 10.1016/j.peh.2015.09.003

[ref61] Pecen E. Collins D. J. Mac Namara Á. (2018). “It's your problem. Deal with it.” performers' experiences of psychological challenges in music. Front. Psychol. 8:2374. doi: 10.3389/fpsyg.2017.02374, PMID: 29422878 PMC5788962

[ref62] Perkins R. Reid H. Araújo L. S. Clark T. Williamon A. (2017). Perceived enablers and barriers to optimal health among music students: a qualitative study in the music conservatoire setting. Front. Psychol. 8:968. doi: 10.3389/fpsyg.2017.00968, PMID: 28701968 PMC5487403

[ref63] Pescosolido B. A. Martin J. K. (2015). The stigma complex. Annu. Rev. Sociol. 41, 87–116. doi: 10.1146/annurev-soc-071312-145702, PMID: 26855471 PMC4737963

[ref65] Picco L. Abdin E. Chong S. A. Pang S. Shafie S. Chua B. Y.. (2016). Attitudes toward seeking professional psychological help: factor structure and socio-demographic predictors. Front. Psychol. 7:547. doi: 10.3389/fpsyg.2016.00547, PMID: 27199794 PMC4842935

[ref67] Qu N. (2022). Individualized assessment and therapeutic intervention for mental health of American postmodern novelists. Occup. Ther. Int. 2022, 1–10. doi: 10.1155/2022/1277121, PMID: 35832100 PMC9259344

[ref68] Renwick L. Pedley R. Johnson I. Bell V. Lovell K. Bee P.. (2022). Mental health literacy in children and adolescents in low-and middle-income countries: a mixed studies systematic review and narrative synthesis. Eur. Child Adolesc. Psychiatry 15, 1–25. doi: 10.1007/s00787-022-01997-6 PMC11032284 35570227

[ref69] Rickwood D. Deane F. P. Wilson C. J. Ciarrochi J. (2005). Young people’s help-seeking for mental health problems. Aust. E-J. Adv. Mental Health 4, 218–251. doi: 10.5172/jamh.4.3.218

[ref70] Rosenbaum D. Weibelzahl S. (2023). Psychological effects of the COVID-19 pandemic on freelance professional musicians. Psychother. Psychosom. Med. Psychol. 73, 321–327. doi: 10.1055/a-2017-5392, PMID: 36863349

[ref71] Rosset M. Baumann E. Altenmüller E. (2022). A longitudinal study of physical and mental health and health-related attitudes among music students: potentials and challenges for university health promotion programs. Front. Psychol. 13:885739. doi: 10.3389/fpsyg.2022.885739, PMID: 35859846 PMC9289676

[ref72] Sampaio F. Gonçalves P. Sequeira C. (2022). Mental health literacy: it is now time to put knowledge into practice. Int. J. Environ. Res. Public Health 19:7030. doi: 10.3390/ijerph19127030, PMID: 35742278 PMC9222847

[ref73] Sandelowski M. Voils C. I. Knafl G. (2009). On quantitizing. J. Mixed Methods Res. 3, 208–222. doi: 10.1177/1558689809334210, PMID: 19865603 PMC2768355

[ref74] Shaw T. A. Juncos D. G. Winter D. (2020). Piloting a new model for treating music performance anxiety: training a singing teacher to use acceptance and commitment coaching with a student. Front. Psychol. 11:882. doi: 10.3389/fpsyg.2020.00882, PMID: 32547438 PMC7270208

[ref75] Smith L. (2021). Review of the current understanding of music performance anxiety, its comorbidities, available treatments and their implementation. Philadelphia, Pennsylvania, US: Temple University.

[ref76] Stuckey M. Richard V. Decker A. Aubertin P. Kriellaars D. (2021). Supporting holistic wellbeing for performing artists during the COVID-19 pandemic and recovery: study protocol. Front. Psychol. 12. doi: 10.3389/fpsyg.2021.577882, PMID: 33613376 PMC7889520

[ref77] Torres-McGehee T. M. Green J. M. Leaver-Dunn D. Leeper J. D. Bishop P. A. Richardson M. T. (2011). Attitude and knowledge changes in collegiate dancers following a short-term, team-centered prevention program on eating disorders. Percept. Mot. Skills 112, 711–725. doi: 10.2466/06.PMS.112.3.711-725, PMID: 21853760

[ref9001] Tricco A. C. Lillie E. Zarin W. O’Brien K. K. Colquhoun H. Levac D.. (2018). PRISMA extension for scoping reviews (PRISMA-ScR): checklist and explanation. Ann. Intern. Med. 169, 467–473. doi: 10.7326/M18-0850 30178033

[ref78] Uriegas N. A. Winkelmann Z. K. Emerson D. M. Moore K. Portillo B. Torres-McGehee T. M. (2024). Treble or trouble: mental health experiences of gender-diverse collegiate marching band artists. J. Athl. Train. 59, 514–521. doi: 10.4085/1062-6050-0367.23, PMID: 38116812 PMC11127677

[ref79] Vaag J. Bjørngaard J. H. Bjerkeset O. (2016). Symptoms of anxiety and depression among Norwegian musicians compared to the general workforce. Psychol. Music 44, 234–248. doi: 10.1177/0305735614564910

[ref80] Van den Eynde J. Fisher A. Sonn C. (2016). Working in the Australian entertainment industry.

[ref9002] Vassallo A. J. Pappas E. Stamatakis E. Hiller C. E. (2019). Injury fear, stigma, and reporting in professional dancers. Saf. Health Work. 10, 260–264. doi: 10.1016/j.shaw.2019.03.001 31497323 PMC6717803

[ref81] Vella S. A. Swann C. Batterham M. Boydell K. M. Eckermann S. Ferguson H.. (2021). An intervention for mental health literacy and resilience in organized sports. Med. Sci. Sports Exerc. 53, 139–149. doi: 10.1249/MSS.0000000000002433, PMID: 32555025 PMC7737879

[ref82] Walker I. J. Nordin-Bates S. M. (2010). Performance anxiety experiences of professional ballet dancers: the importance of control. J. Dance Med. Sci. 14, 133–145. doi: 10.1177/1089313X1001400402, PMID: 21703084

[ref9003] Wang X. Liang W. Liu J. Zhang C. Q. Duan Y. Si G.. (2022). Further examination of the psychometric properties of the multicomponent mental health literacy scale: evidence from Chinese elite athletes. Int. J. Environ. Res. Public Health. 19:12620. doi: 10.3390/ijerph191912620 36231919 PMC9566777

[ref83] Washington-Simon K. T. (2024). All Eyez on U: Exploring aspects of mental health and well-being with hip hop recording artists-a need assessment (Doctoral dissertation, National University).

[ref84] Williamon A. Thompson S. Lisboa T. Wiffen C. (2006). “Creativity, originality, and value in music performance,” in Musical creativity. Psychology Press. p. 177–196.

[ref86] Žilinskas E. Lesinskienė S. (2023). Suicide literacy and attitudes toward psychological help-seeking: a cross-sectional study of students. J. Int. Med. Res. 51, 1–11. doi: 10.1177/03000605231172452, PMID: 37194200 PMC10192667

