# Supplementary material for: A scoping review of mental health literacy in performing and creative artists: identifying current gaps and future directions
Source: Front Psychol. 2025 Aug 22;16:1329029. doi: 10.3389/fpsyg.2025.1329029 (PMC12412305; doi:10.3389/fpsyg.2025.1329029)
Supplement: Supplementary file 1 [file Supplementary_file_2.docx]

**Appendix 2. MEDLINE search strategy**

1. (artist or artists or painter* or musician* or photographer* or dance* or pianist* or violin* or visual artist* or writer* or poet* or screenwriter* or illustrator* or graphic designer* or sculptor* or painter* or acrobat* or comedian* or singer* or art* teacher or art* student* or playwright* or photographer* or performer*).tw,kf.
2. consumer health information/ or health literacy/
3. Patient Education as Topic/
4. (health promotion or health education or health curriculum or mental health awareness or health knowledge or attitude* to health or seeking help or help seeking behaviour*).tw,kf.
5. 2 or 3 or 4
6. mental health/ or exp mental disorders/
7. ((literac* or literate or educat* or promotion or awareness or stigma*) adj5 (mental health or wellness or wellbeing or mental disorder* or depress* or bipolar* or anxiet* or anxious* or stress* or PTSD or post-traumatic stress disorder*)).tw,kf.
8. 5 and 6
9. 7 or 8
10. 1 and 9
11. remove duplicates from 10
