# Supplementary material for: A scoping review of mental health literacy in performing and creative artists: identifying current gaps and future directions
Source: Front Psychol. 2025 Aug 22;16:1329029. doi: 10.3389/fpsyg.2025.1329029 (PMC12412305; doi:10.3389/fpsyg.2025.1329029)
Supplement: Supplementary file 2 [file Supplementary_file_3.pdf]

Appendix 3. PRISMA 2020 flow diagram

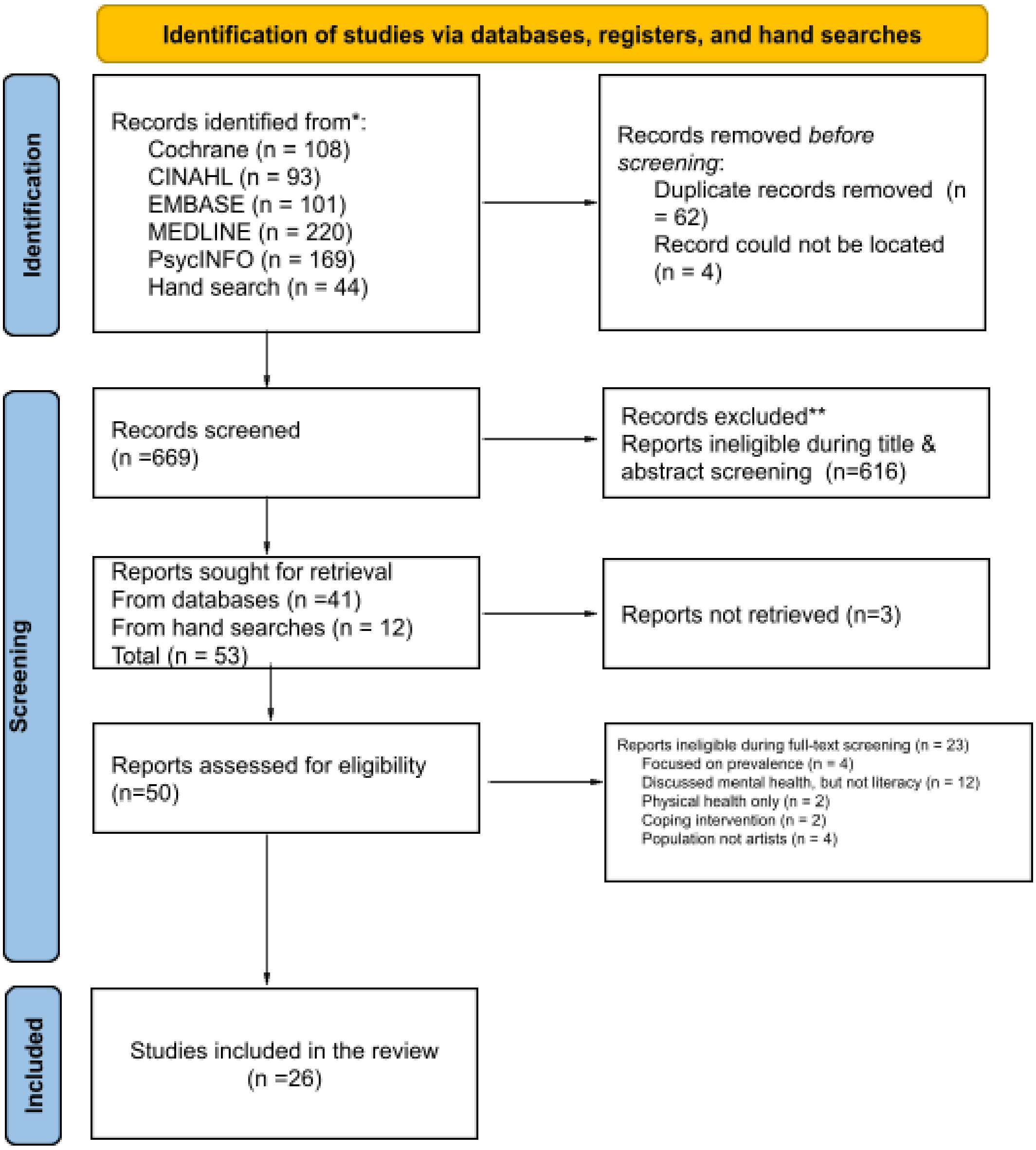

*Consider, if feasible to do so, reporting the number of records identified from each database or register searched (rather than the total number across all databases/registers).

**If automation tools were used, indicate how many records were excluded by a human and how many were excluded by automation tools.

From: Page MJ, McKenzie JE, Bossuyt PM, Boutron I, Hoffmann TC, Mulrow CD, et al. The PRISMA 2020 statement: an updated guideline for reporting systematic reviews. BMJ 2021;372:n71. doi: 10.1136/bmj.n71
