# Supplementary material for: A scoping review of mental health literacy in performing and creative artists: identifying current gaps and future directions
Source: Front Psychol. 2025 Aug 22;16:1329029. doi: 10.3389/fpsyg.2025.1329029 (PMC12412305; doi:10.3389/fpsyg.2025.1329029)
Supplement: Supplementary file 3 [file Table_1.docx]

Supplementary Table 1

| **Author (Year),**  **Setting,**  **Country of Publication** | **Sample Size** | **Sample Characteristics** | **Study design** | **Purpose** | **Data collection methods** | **Main findings and definition of mental health literacy (if applicable)** |
| --- | --- | --- | --- | --- | --- | --- |
| Atkins (2009),  7 music conservatories located in the UK,  United Kingdom | N = 46 | 26 male and 20 female participants with a mean age of 22. Students were studying at the undergraduate and postgraduate level. 27 students had experienced an injury, 19 had not and 15 were currently dealing with an injury. | Qualitative | To examine the health and well-being of students enrolled in music conservatories, and to examine resources currently available to students who may be experiencing an injury. | Case studies were used to compile data on conservatories. Students were asked to complete a self-reporting questionnaire. | All seven conservatories provided an array of resources for students who may be experiencing injuries. Yet, not all students could identify these resources, with only 22% noting that they felt the conservatories were adequately assisting students with their health. Mental health literacy is not defined in this study. |
| Barnett et al. (2019),  Traveling art exhibition targeted towards rural towns,  Australia | N = 168 | N = 23 (artists who submitted their work and have been affected by mental illness), n = 145 (visitors to the art exhibition) | Mixed | To understand  the experiences of artists who struggle with mental health issues and to  reduce stigma towards mental health in rural communities | Data were collected using semi-structured interviews and thematically analysed. This was augmented by survey data (n=145) from visitors to the exhibition over 3 successive years. | The community art exhibitions were shown to have positive benefits for artists and the community. Artists found their knowledge of mental health issues and services increased through the exhibitions. One artist noted a reduction in shame of their mental health problems.  This study defined mental health literacy as “knowledge of mental health issues and services” |
| Barton & Feinberg (2008),  A private university located in the midwest of the United States,  United States | N = 26 | Freshmen music major students | Quasi-experimental | To evaluate the implementation of a music and health course within an existing music program, and to evaluate students’ knowledge of health promotion and injury prevention. | Demographic form, course content questionnaire and self-assessment questionnaire using a 5-point Likert scale. | 20 students indicated having no formal instruction on injury prevention. Over 40% of participants indicated that they infrequently or never use health promotion techniques. Authors recommend that the intervention should change to target attitudinal change. Mental health literacy is not defined in this study. |
| Bartos et al. (2022),  Higher education music students in Granada, Spain,  USA | N = 26 | CRAFT (musicians’ health and wellness program) participants who were full-time higher education student musicians, 18 years or older | Qualitative | To gain a greater understanding of participants’ CRAFT-based practice implementation experience and their perceived benefits derived from it during the lockdown. | An open-ended question in an online questionnaire asking which particular practice or practices participants had implemented and describe their own experience with it and the impact they had on their physical, mental, and emotional state. | Participants perceived many benefits including enhanced conscious awareness, emotional self-regulation, mind clearance, transforming attitudes, and resilience. Mental health literacy is not defined in this study. |
| Cardinal & Hilsendager (1997),  Literature review using the following databases: Educational Resources Information Center [ERIC], Psychlit, and SPORT Discus. Also interviewed five professionals,  United States | Total N = 11.  N = 5 for initial interviews/  discussions.  For stage 1: N = 3 (out of the first 5).  For stage 2 of the draft: 3 external experts  For draft 3: another different 3-member panel. | For initial interviews/informal discussion and stage 1 of the draft: professionals in  the fields of dance medicine, dance science, somatics, wellness, and education. For stage 2: panel of expert educators/scholars in dance education, dance medicine, dance science, and/or somatics. For stage 3: 3-person panel of experts | Qualitative | 1. To extract from the literature in dance medicine, science, somatics, and related fields those curricular offerings and implementation strategies that comprise “dance wellness” and  have been recommended by experts  for study by college and university  dance students and 2. To synthesize  the information into a curricular  model for dance wellness education. | Interviews, informal discussion, evaluation form | Identified a definition of dance wellness, objectives and functions of dance wellness, ten major components, four implementation strategies, and other extra programs for dance wellness. While mental health literacy is not defined, dancer wellness (encompassing mental health) is. |
| Cardinal et al.(2020),  4-year institutions in the U.S. with undergraduate or graduate programs listed in the Dance Magazine College,  United States | N = 199 | Higher education dance administrators at 4-year institutions (selected by stratified random sampling) | Cross-sectional study | To explore the inclusion of dancer  wellness education in U.S. colleges and  universities. | Survey consisting of 5 sections: 1) dance program description, 2) dancer wellness program(s) offered, 3) employment of dancer wellness specialists, 4) dancer wellness curriculum, 5) inclusion of seven types of dance or wellness supplementary programs | 60% offered a formal dancer wellness program and 70% had at least one dancer wellness specialist on faculty. The top five topics were anatomy, kinesiology, somatics, dance conditioning, and dance injuries. Mental health literacy is undefined but the study does discuss the status of dancer wellness education, including psychology. |
| Clements-Cortés et al. (2023)  2 music schools in Ontario | N = 15 | 13 female participants, 1 male and 1 non-binary. Participants ranged in age from 21 to 33 years old. Participants’ principal instruments included piano (n = 7), voice (n = 4), saxophone (n = 2), clarinet (n = 1), and flute (n = 1). Participants’ years of study in music education ranged from 1 to 7 years | Sequential mixed methods | To explore the benefits of participating in group music psychotherapy, to determine helpful techniques for reducing music performance anxiety, and to identify implications for music students on including music psychotherapy as an intervention for MPA | Intake questionnaire, the Spielberger’s State-Trait Anxiety Inventory (STAI) was administered pre and post-study, Likert scale anxiety ranking was administered pre and post-study, and a post study questionnaire. | Results from the Anxiety Questionnaire showed that participants reported a significant decrease in anxiety after participating in the music psychotherapy sessions. 63% of the participants stated in post-questionnaires that the music psychotherapy sessions helped them to better manage their MPA. |
| Ginsborg (2012),  Multiple studies,  N/A | N/A (multiple studies included, not all sample sizes are given) | N/A | Literature review | To review existing literature that explores occupational injuries and mental well-being experienced by artists, and general knowledge and frameworks used by artists for promotion of well being. | N/A | Results of the literature review show that although playing a musical instrument is shown to improve health, professional musicians exhibit an irregular amount of distress in comparison to the general population. Musicians also seemed to lack prioritization of physical and mental well-being. Mental health literacy is not defined in this study. |
| Griffith (2023)  Study 1: Instagram  United States | Study 1: N = 17  Study 2: N = 318Study 1: N = 17  Study 2: N = 318 | Study 1: participants were at least 18 years old, fluent in English and lived in the United States. Must have at least 100 Instagram followers and have a history of posting about a personal mental health diagnosis on Instagram in an artistic format.  Study 2: participants were recruited online through Amazon Mechanical Turk, fluent in English and lived in the United States | Mixed Methods  (Study 1: semi-structured interviews; Study 2: Randomized Controlled Trial) | Study 1: To gain a better understanding of artists’ lived experiences, to understand their motivation to post their art online and their identities as both artists and people with mental illness.  Study 2: To compare the effectiveness of viewing artwork by people who experience mental illness compared to artwork that did not represent mental illness. Artwork from Study 1 was stimuli for participants in Study 2 | Study 1: Semi-structured interviews  Study 2: Prejudice towards People Experiencing Mental Illness Scale (Kenny et al., 2018) and the Empathy State Scale (Shen, 2010) | Participants (Study 2) in the intervention groups reported more change in their mental health awareness and sympathy for people with mental health illness. Self-reported prejudice was also lower in post-test scores. |
| Guptill et al. (2022),  Post-secondary schools in Canada, USA, South Africa, Australia, New Zeland, and United Kingdon,  Switzerland | N = 549  (n=328 for the retest of MHL-Q19 only) | Post-secondary music students from Canada  (n=253), the United States (n=138), South Africa (n=43),  Australia (n=57), New Zealand (n=31), and the United Kingdom  (n=27). | Quantitative | To evaluate the validity and reliability of the MHL-Q19. | Musicians’ Health Literacy  Questionnaire, MHL-Q19 | The tool showed acceptable reliability. The MHL-Q19 questions mostly map onto the intended domains of the health literacy conceptual framework. Musicians’ health literacy and general health literacy were shown to have weak, but present, overlap. The internal consistency of the MHL-Q19 is high but its test–retest reliability was lower than expected. While mental health literacy is undefined, health literacy “entails  people’s knowledge, motivation and competences to access,  understand, appraise, and apply health information in order  to make judgments and take decisions in everyday life  concerning healthcare, disease prevention and health  promotion to maintain or improve quality of life during the  life course.”  (Sorensen et al., 2012) |
| Harrison et al. (2019),  National ballet company (location not specified – Australia?),  Netherlands | N = 5  (N = 3 male, N = 2 female) | Professional ballet dancers between 24-30 years old. N=1 senior artist, N=2 soloists, N=1 coryphee, and N=1 corps de ballet | Mixed methods | To test the use of a monitoring app designed for professional dancers to measure self-reported wellness constructs | Wellness and ballet-specific activity questionnaires daily through the  wellness App.  Focus group interview 1 month after data entry period. | Dancers reported that the App enhanced awareness of both physical and psychological well-being. Two dancers regularly reviewed their fatigue and self-reported sleep quality and quantity. Mental health literacy is not defined but the study discusses health monitoring (including mental aspected) and developing self-awareness |
| Kaufman et al. (1996),  School of American Ballet, USA,  United Kingdom | N = 39 | Female dancers between ages of 13-17; 8 completed the study, 31 did not | Quantitative | To determine if education, with counselling and physical therapy if needed, could decrease the incidence of stress fractures and dieting behaviours. | Physical and medical assessment, food intake history, activity level records, EAT26 questionnaire, Offer Self-Image Questionnaire (OSIQ) | The study suggests that subjects who completed the study were naturally thinner, more premenarchal, had less dieting behaviour at baseline, and were further from their recommended weight than those who did not. We also found that their weight approached ideal, even though dieting behaviour increased. High-risk students appear to be those nearer their ideal weight. Within the group that did not complete the study, we found a higher level of aberrant eating behaviours.  This study does not define mental health literacy but examines the mental health education program |
| Mathisen et al. (2022)  University students in the arts,  Switzerland | N=164 | N=125 dance students, N=39 arts and craft students (references) | Longitudinal study with parallel group design | To examine an intervention designed to increase mental health literacy, enhance nutritional knowledge, reduce symptoms and effects of low energy availability, and strengthen understanding of sports nutrition and recovery strategies, in dance students. | Eating Disorder Examination questionnaire, the Low Energy Availability in Females questionnaire, the Hopkins Symptom Check List, and questions on mental health literacy, sports nutrition, and recovery knowledge. | Dancers had significantly lower mental health literacy at baseline. Dance students achieved sustained improvements in mental health  and nutrition knowledge and temporary improvements in driven exercise (i.e., performing exercise because of a compulsive drive). No other benefits were identified from the intervention. While mental health literacy is not explicitly defined, the intervention is designed to improve mental health literacy consisting of understanding physical and mental health and performance effects as well as nutrition and recovery strategies |
| Moore et al. (2024)  United States  Collegiate marching bands | N = 534 | Members of collegiate marching bands across the United States between the ages of 18-26. | Observational study with a cross-sectional design, quantitative | To examine the perceived barriers to mental health support experienced by marching band artists, as well as to explore barriers to accessing mental health care between genders. | Questionnaires including Demographic questionnaire, the Barriers to Help Seeking Checklist, Attitudes Toward Seeking Professional Psychological Help-Short Form Scale (ATSPPH-SF) and the Mental Help Seeking Attitudes Scale (MHSAS) | The greatest barrier reported was lack of time to seek services (69.1) followed by unavailability of services during free time (47.6%). Overall, more positive attitudes towards seeking mental health support was reported. |
| Perkins et al. (2017),  6 music  conservatories in the United Kingdom,  United Kingdom | N = 20 | Musicians who  either recently graduated or are currently studying at a music conservatoire | Qualitative | To examine the  demands musicians experience and to explore the attitudes musicians have  towards mental health and well-being | Individual semi-structured interviews (on perceived enablers and barriers and personal attitudes) | 90% of participants reported that health and well-being were important to them as musicians. 95% of participants reported self-awareness towards mental health and well-being. 55% of students reported a lack of adequate support for their mental health and well-being. Mental health literacy is not defined in this study. |
| Qu (2022),  American postmodern novelists,  US | N = 30 | Members who met the  experimental intervention were divided into experimental group and control group, and the experimental group received a  30-day reception music therapy intervention. | Quantitative | The application of art therapy in the  mental health education of American postmodern novel creators | Questionnaires; quasi-experimental | Receptive music therapy intervention has a significant effect in relieving the anxiety of American postmodernist novel creators. It also plays a positive role in helping American postmodernist novel creators better adapt to study life, and build good emotional psychology and interpersonal relationships. Mental health literacy is not defined in this study. |
| Rossett et al. (2022),  Music students in postsecondary institution,  Germany | N = 205 | Investigated mental and physical health status, health-related attitudes, knowledge, skills, behaviours, and coping strategies of music students at the beginning of their first year. Next, they analyzed differences between performance and music education majors and between students playing different main instruments. In a subsample (n = 62), we additionally analyzed changes between the beginning of the music students’ first and the end of their second semester, also depending on whether they attended courses on musicians’ health | Quantitative | To examine health status, health-related attitudes, behaviours, knowledge, skills, and coping strategies of students at the beginning of their education at a music university and at the end of their second semester. | Questionnaire | After two semesters, students attending courses on musicians’ health showed increased knowledge and skills regarding different aspects of musicians’ health.  Mental health literacy is not defined in this study. |
| Shaw et al. (2020),  Private music studio at a university in the United Kingdom,  United Kingdom | N = 1 | The participant is a music theatre major who had reported problematic levels of music performance anxiety (MPA) | Mixed | To examine the impact of an Acceptance and Commitment Coaching (ACC) coaching model on a student with problematic levels of MPA | Coaching and a semi-structured reflection interview | The participant showed greater ability to mitigate symptoms of MPA after being exposed to ACC coaching sessions. Mental health literacy is not defined in this study. |
| Smith (2021)  Multiple studies  United States | N/A (multiple studies included, not all sample sizes are given) | Review of the current understanding of music performance anxiety, its comorbidities; available treatments and  their implementation | Disssertation (literature review) | To provide a model for a  physical and mental health plan designed for students, educators, and schools | N/A | Examined available treatments for general mental health and music performance anxiety. Investigated mental health services and courses offered in five institutions Mental health literacy is not defined in this review. |
| Stuckey et al. (2021),  Elite-level circus training school based in Montreal, Canada,  Canada | N = 110 | Circus arts students enrolled in the college program | Quantitative | To examine variation in psychological characteristics among circus arts students within the training year, and to compare these characteristics with pre-pandemic data. | Questionnaire | Since this is a longitudinal study design, the results are currently being analyzed. The results will aim to support the implementation of holistic and resilience-promoting programs to promote the well-being of circus arts students. Mental health literacy is not defined in this study. |
| Torres & Green (2011),  Collegiate female dancers,  United States | N = 32 | The experimental group received an intervention during the spring  semester, consisting of an educational program to prevent eating disorders. They were compared to the control group. | Quantitative | To compare two groups of students (experimental versus control) to determine the effectiveness of an educational program. | Quasi-experimental | There was a statistically significant increase in scores on nutritional and overall eating disorder knowledge in the intervention group compared to the control group. Mean scores on depression, drive for thinness, body dissatisfaction, and maturity fears decreased in the intervention group. Mental health literacy is not defined in this study. |
| Uriegas et al. (2024)  Collegiate Marching Bands  United States | N = 78 | Gender diverse (GD) collegiate marching band artists between the ages of 18-26, currently enrolled in a university or college. | Cross-sectional, quantitative | To examine the risks of anxiety and depression amongst gender diverse marching band artists and to explore barriers and attitudes towards accessing mental health support. | Online surveys using a Demographic survey, State-Trait Anxiety Inventory, Center for Epidemiologic Studies Depression Scale, Barriers Towards Seeking Help Checklist, Attitudes Toward Seeking Professional Psychological Help Scale-Short Form, Mental Help Seeking Attitudes Scale. | Overall, the majority of the marching band artists (78.2%) were considered at risk for both depression and anxiety. The most commonly reported barrier to accessing mental health support was lack of time (82.1%). The general attitude towards seeking mental health support was positive. |
| Walker & Bates, (2010),  Elite dancers in one company,  UK | N = 15 | Elite ballet dancers | Qualitative | To explore ballet dancers' experiences of performance anxiety in relation to: 1. symptom type, intensity, and directional interpretation; 2. experience level (including company rank); and 3. self-confidence and psychological skills. | Individual interviews | Most dancers recognized that some anxiety was beneficial, especially in relation to somatic anxiety. Principal dancers experienced higher intensities of performance anxiety than corps de ballet members. Feeling out of control emerged as a major theme in both the experience of anxiety and its interpretation. Mental health literacy is not defined in this study. |
| Washington-Simon(2024)  Across the country  United States | N = 5 | Hip hop artists who have personally experienced mental health challenges throughout their career. | Qualitative | To explore the need for mental health and well-being amongst Hip Hop recording artists, identify barriers to seeking mental health support, and any coping strategies currently being implemented. | Purposive sampling, semi-structured interviews | Hip Hop artists reported living in a state of constant demand and perseverance. This lead to an increase in mental health challenges. The artists reported a lack of perceived sympathy from the general public, decreasing the likelihood of seeking out mental health support. Artists also reported perceived stigma against Hip Hop artists. |
| Williamon & Thompson (2006),  Royal College of Music in London, United Kingdom,  United Kingdom | N = 63 | Music major undergraduates enrolled in an introductory seminar on music and health | Qualitative | To examine the awareness and incidence of physical and mental health problems relating to music performance | Individual interviews | Students usually go to their primary instrumental teachers rather than healthcare practitioner when concerned about their health. A curriculum initiative is recommended in helping music students increase their knowledge and awareness of mental health. Mental health literacy is not defined in this study. |
| Žilinskas & Lesinskienė (2023)  Higher education institutions  Lithuania | N = 2004  N = 172 art students | Students currently enrolled in universities across Lithuania | Cross-sectional, quantitative | To determine students’ level of knowledge of suicide and and attitudes towards seeking professional help. To assess students’ suicide literacy. | Questionnaires using the Literacy of Suicide Scale, the Attitudes Toward Seeking Professional Psychological Help Scale and the Suicidal Ideation Attributes Scale | Suicide literacy was highest amongst biomedical students as well as female students. Both groups also demonstrated the most positive attitudes towards seeking mental health support. Art students expressed the highest levels of suicide ideation. |
